# SoyDBean: a database for SNPs reconciliation by multiple versions of soybean reference genomes

**DOI:** 10.1038/s41598-023-42898-1

**Published:** 2023-09-21

**Authors:** Yejin Lee, Dong U Woo, Yang Jae Kang

**Affiliations:** 1 Division of Bio and Medical Bigdata Department (BK4 Program), Gyeongsang National University, 501, Jinju-daero, Jinju-si, Gyeongsangnam-do 52828 Republic of Korea; 2 Division of Life Science Department, Gyeongsang National University, Jinju, Republic of Korea

**Keywords:** Databases, Data publication and archiving

## Abstract

Due to the development of sequence technology and decreased cost, many whole genome sequences have been obtained. As a result, extensive genetic variations have been discovered from many populations and germplasms to understand the genetic diversity of soybean (*Glycine max* [L.] Merr.). However, assessing the quality of variation is essential because the published variants were collected using different bioinformatic methods and parameters. Furthermore, despite the enhanced genome contiguity and more efficient filling of “N” stretches in the new reference genome, there remains a dearth of endeavors to verify the caliber of variations present in it. The primary goal of this research was to discern a dependable set of SNPs that can withstand reconciliation across multiple reference genomes. Additionally, the investigation aimed to reconfirm the variations through the utilization of numerous whole genome sequencing data obtained from publicly available databases. Based on the result, we created datasets that comprised the thoroughly verified SNP coordinates between the reference assemblies. The resulting “SoyDBean” database is now publicly accessible through the following URL: http://soydbean.plantprofile.net/.

## Introduction

Soybean (*Glycine max* [L.] Merr.) is a legume that is one of the most important crops for animals and humans, owing to its high protein and oil content. It can also hold atmospheric nitrogen through symbiosis with microorganisms^1^. *G. max* is known to have been domesticated from East Asia around 7000–9000 years ago^2^. With the development of Mendel’s laws of heredity and the advancement of our understanding of plant genetics, molecular breeding techniques became popular^3^. Multi-omics breeding methods are now used in many experimental breeding programs, along with recent improvements in the methods of phenotypic and molecular observation on soybean plants^4^.

The utilization of next-generation sequencing (NGS) technology has revolutionized the acquisition of complete genome sequences and the generation of high-throughput sequencing data for researchers, making it a cost-effective and effortless process. Moreover, the availability of vast amounts of Next-Generation Sequencing (NGS) data enables high-depth observations for each base, ensuring the accuracy of the data, and allowing for the identification of sequence variations at the single base level^5^. To comprehend the genetic diversity of crops, nucleotide variations were detected by this technology in numerous populations utilizing the reference genome^6^. In soybean, vast amounts of single nucleotide polymorphisms (SNPs) were generated through various studies that aimed to compare genetic variation in wild and cultivated soybeans and identify allele diversity specific to wild soybeans^7^, identify genetic information available for soybean breeding^8^, study the genetic diversity and structural properties of soybeans^9,10^, construct a haplotype map using whole genome sequence data^11^, and etc.

The creation of these large datasets was driven by the fact that a consolidated matrix of accumulated variants encompassing a broad spectrum of genetic diversity in both wild and cultivated soybeans would provide immensely informative insights into understanding allelic diversity that may be linked to useful phenotype^11,12^. However, integrating individual research variants data into these large datasets is not straightforward due to the differences in variant calling progress, tools, and data filtering employed.

SNP calling based on read mapping to the reference genome involves several bioinformatic steps. Typically, NGS reads are sequenced and then aligned to the reference genome. Subsequently, a series of quality control processes, including the removal of duplicated and poor-quality reads, are performed. After these steps, the reads are realigned, and variants are detected^13^. Since there is no gold standard for this pipeline, various pipelines, and filtering criteria have been employed to create different published datasets (Supplemental Table 1).

Moreover, as sequencing technologies and analytical techniques continue the improve, reference genomes are frequently updated^14^. The first reference genome assembly for *G. max* was constructed in 2010 using the whole-genome shotgun sequencing method, and it contained 17.7 Mb in 1148 unmapped scaffolds and 950 Mb in 20 chromosomes^15^. Subsequently, a Wm82.a2.v1 assembly of *G. max* was published, which consisted of 20 chromosomes totaling 949.2 Mb and 1170 unmapped scaffolds with an N50 value of 29.3 Mb. This new assembly was able to identify the mislinked scaffold, realign it, and fill the contig gap with the available linkage genetic map^16^. Such advancements in reference genome assembly and annotation provide researchers with improved resources for analyzing genomic data. The presence of discordance in genetic variation calls between old and new references poses a challenge in determining the reliable version. This challenge arises from the intricate nature of the source and the quality of the raw data, as well as the assembly algorithm. These factors encompass numerous potential sources of errors, even when the raw data and algorithm are comparatively more recent than others^17^.

Various remapping tools, such as LiftOver^18^, Crossmap^19^, and NCBI-remap [available at ncbi.nlm.nih.gov/genome/tools/remap], have been developed to transfer physical positions across different genome versions. Nonetheless, the resulting variation from the recently built genome has not undergone comprehensive validation, leading to discrepancies in the number of SNPs observed between the old and new reference genomes^20^. Therefore, it is essential to have a set of SNPs that can be commonly extracted from any reference version and stored in a more transferable format, making it simple to assign SNP positions related to the reference version^21^. To address this issue, the SNP database process has been carried out with tools such as GATK, especially for human genome analysis. Central to this validation process is the availability of a comprehensive and validated reference dataset, such as dbSNP^22^. A human genome SNP database was created to verify the dependability of SNPs, but its availability for the soybean genome remains unavailable at present.

In this study, our objective was to identify comparable and reliable SNPs by consistently detecting SNP calls from two different version of the *G. max* reference genomes. In order to establish the reliable SNPs, we employed a two-step process. Firstly, we used the flanking sequences of SNPs from each reference genome and compared them to each other’s reference genome using mapping tools. This resulted in a preliminary list of comparable SNP positions between the two genomes. Secondly, we re-mapped reads from various soybean accessions to both reference genomes and evaluated the preliminary list of comparable SNP positions. Through this process, we identified SNPs that were consistently and reliably called, regardless of the reference genome versions. This database allows for the filtration of SNPs, resulting in a reliable set of SNPs that can be integrated into the GATK pipeline.

## Methods

### Data sources and calling SNPs

**Figure 1:**
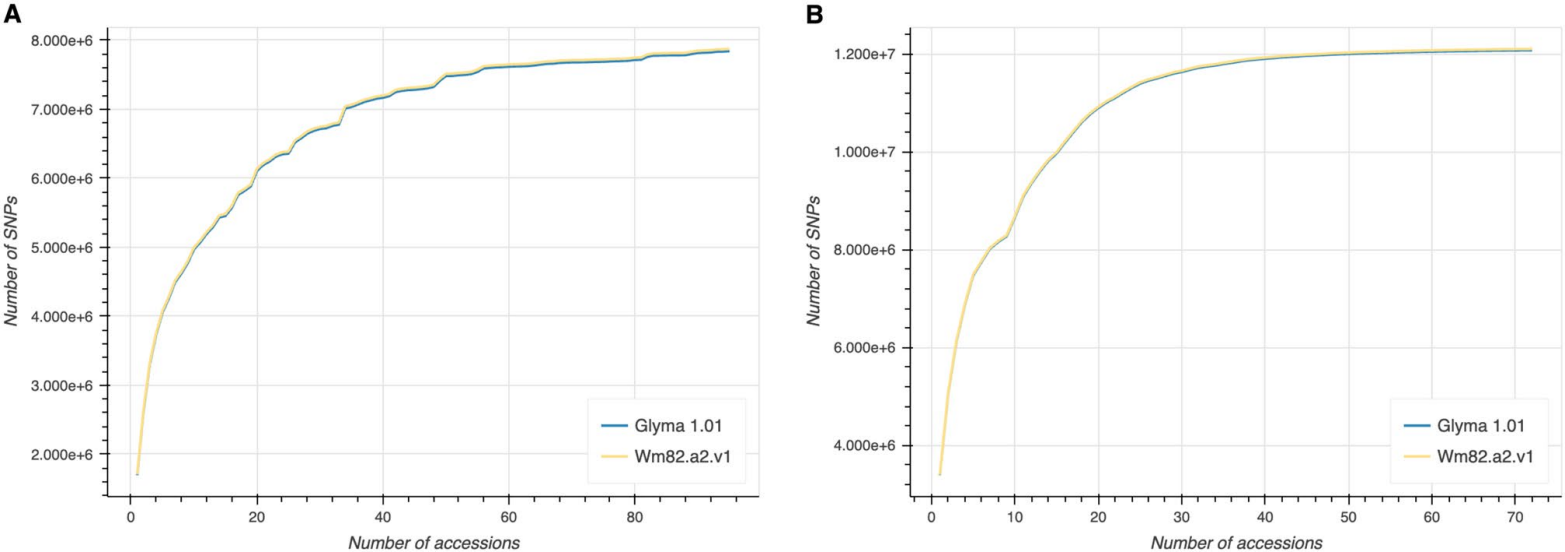
The count of SNPs identified against the reference genome versions, Wm82.a2.v1 and Glyma 1.01, using short read mapping with increasing numbers of accessions for each of the two groups: (**A**) *G. max* and (**B**) *G. soja*.

The SNP datasets used in the SoyDBean database consist of subpopulations of 95 cultivated soybeans (G. max) and 72 wild soybeans (G. soja), collected from six countries (South Korea: 146; China: 11; Japan: 6; USA: 2; Canada: 1; Sweden: 1, PRJNA555366) to obtain a vast amount of variant data (Supplemental Table 3). The reads of 167 accessions were mapped to each reference genome (Glyma 1.01 and Wm82.a2.v1) using the Burrow-Wheeler Aligner (BWA)^23^. A duplicate was removed using samtools^24^ and then sorted based on coordinates using the sambamba sort^25^. Finally, the SNPs were identified and called using bcftools^26^. After SNP calling, it was filtered on the overall quality (QUAL) > 30, the mapping quality > 30, the minor allele frequency (MAF) > 0.02, and the fraction of missing genotype < 0.05 and stored in Variant Call Format (VCF). Through 183 soybean accessions (102 *G. max* and 81 *G. soja*), we detected 12,447,986 SNPs in Glyma 1.01 and 12,473,679 SNPs in Wm82.a2.v1. To determine whether a sufficient number of SNPs had been identified, we examined the increase in the number of SNPs with the addition of accessions cultivated soybean and wild soybean separately. We observed that the number of SNPs did not significantly increase beyond 90 accessions for *G. max* and 50 accessions for *G. soja*, indicating that an adequate number of SNPs had been discovered (Fig. 1). The process of variant calling was conducted by this pipeline (https://github.com/yeah-zin/soydbean-variantCalling-pipeline).

### Identify the reliable SNP positions for each version

**Figure 2:**
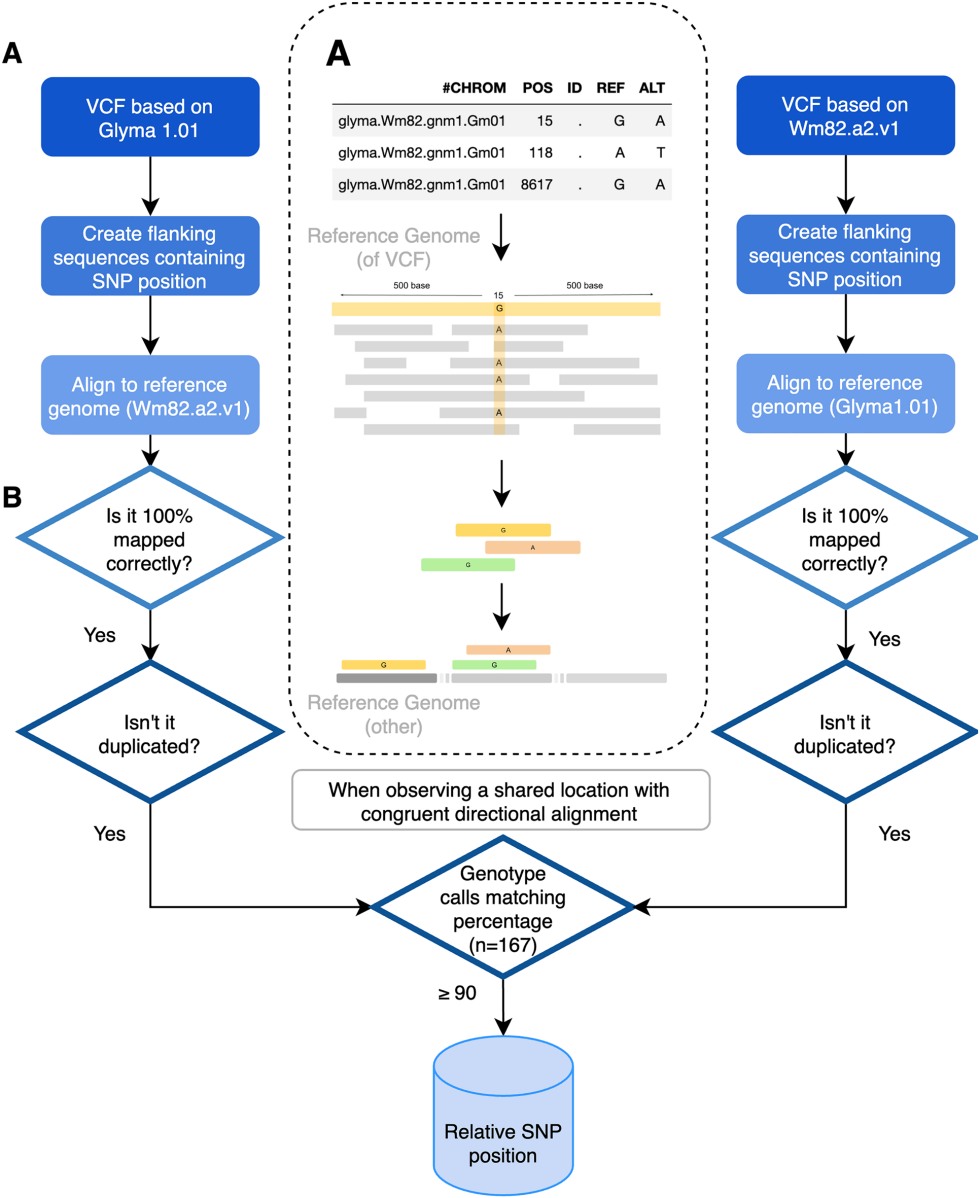
Schematic overview of database construction (**A**) SNP flanking sequence extraction process from the reference genome based on the SNP positions. (**B**) The verification process for identifying a reliable SNP through stringent cut-off criteria and reconciliation of genotype using 167 accessions.

The overall workflow of reconciliation of SNPs for each version is shown in (Fig. 2). We extracted flanking sequences (containing 500 bp on both sides of the SNP positions, 1001 bp) of SNP positions in each reference genome version. A total of 12,432,846 reads based on Glyma 1.01 and 12,458,909 reads based on Wm82.a2.v1 were stored in Fasta format. The flanking sequences of SNPs were mapped 100% identity using the BWA-mem algorithm to another reference genome to verify that the sequences containing SNPs are valid between the references (Supplemental Table 3). To increase reliability, it was filtered by whether sequences were mapped 100% identity and whether they were mapped to multiple positions. Most flanking sequences (92.52% Glyma 1.01 to Wm82.a2.v1 and 92.58% Wm82.a2.v1 to Glyma 1.01) were successfully mapped to each reference genome. Other sequences were not matched (7.48% and 7.41%) or duplicated mapped (0.004% and 0.005%). As a result, the reliable SNP positions that are represented by the conservation of the flanking sequence between the references were 11,502,261 SNPs in Glyma 1.01 to Wm82.a2.v1 and 11,534,444 SNPs in Wm82.a2.v1 to Glyma 1.01. We determined that 11,314,158 SNPs are reliable through a process of mutual confirmation between multiple references.

### Comparing genotypes in VCFs across different reference versions

**Figure 3:**
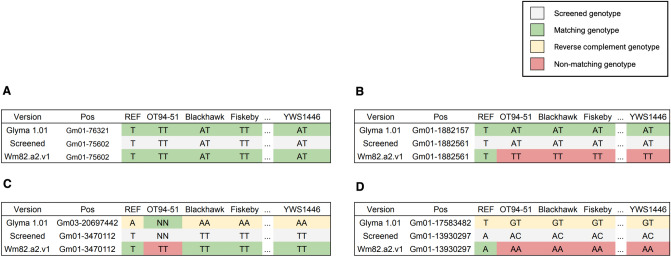
Example of comparison of genotypes for each version of VCF. (**A**) flanking sequences were mapped forward, and the concordance rate was ≧90% (87.6% of all SNPs). (**B**) it was mapped forward, and the concordance rate was < 90% (0.46% of all SNPs). (**C**) it was mapped reverse complement, and the concordance rate was ≧90% (11.8% of all SNPs). (**D**) it was mapped reverse complement, and the concordance rate was < 90% (0.13% of all SNPs).

**Figure 4:**
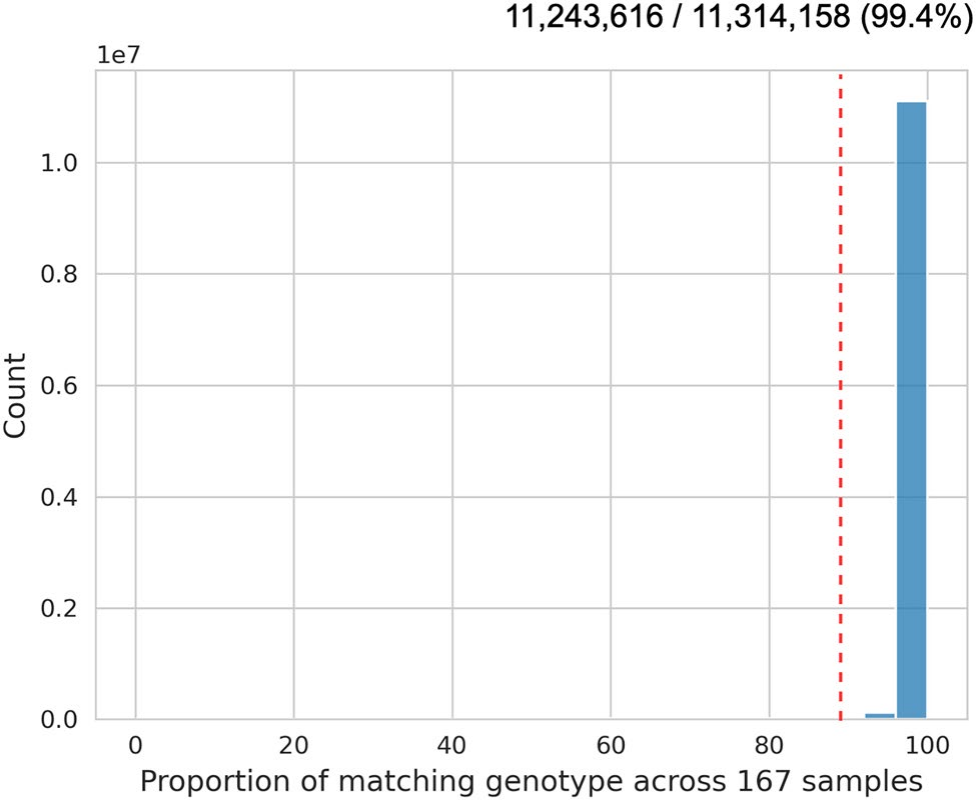
Histogram of genotype match ratios of SNPs through mutual comparison between references using paired-end short reads from 167 soybean accessions.

We used paired-end short reads from 167 soybean accessions to confirm the reliability of transferable SNPs identified through mutual comparison between references. We compared each genotype calls at a position that was assessed to be the same SNP and found that 99.4% of the genotype at the reliable 11,243,616 SNP position agreed between the references in more than 90% of 167 soybean accessions (Fig. 3). We found that a small portion (0.6%) of SNPs were not consistently called between the references when analyzing 167 soybean accessions (Fig. 4). This may be due to unresolved duplicate mapping results or the specific base composition at these SNP positions, which could cause noise in the genotyping process. Utilizing a filter of 90% match rates on 167 accessions, we successfully identified 11,243,616 reliable SNPs, which enabled us to construct a comprehensive database containing information on reference version-wise SNP positions.

### Signature of flanking sequences

**Figure 5:**
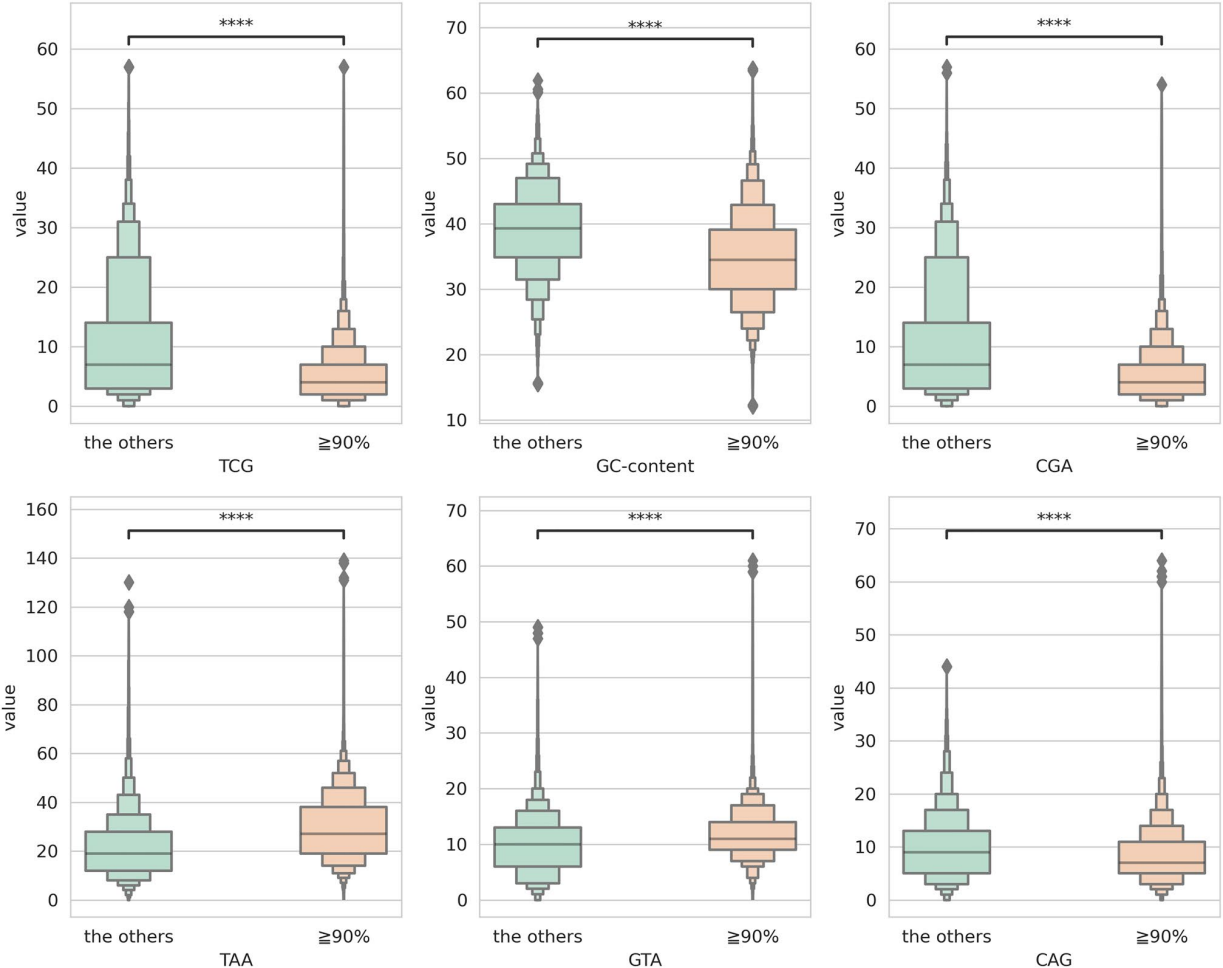
Significant 3-mers in SNP flanking sequences for the validation of some SNPs (≧90%: genotype match rates ≧90%; the others). The y-axis represents the count values of 3-mer frequencies or the ratio of GC-content. A statistical analysis using a t-test was conducted to compare ≧90% and the others’ SNP positions. ****p ≤ 0.0001.

To understand the reasons for the differences in genotype calls of certain SNPs using 167 accessions, we conducted a K-mer analysis on the flanking sequences of the SNPs. We utilized the 3-mer frequency, SNP position, and GC content as indicators to differentiate between genotype calls matching and non-matching sequences. To statistically compare genotype calls match rates ≧ 90% group and the others group, then performed t-test (Fig. 5). Our findings suggested that 3-mers containing either “C” or “G” exhibited significant discrepancies between the two groups when examining SNP flanking sequences. Those with a low number of such 3-mers were more likely to be consistent with genotype calls excluding “TAA”, whereas those with high numbers of “TCG” 3-mers were more likely to be inconsistent. This K-mer analysis provided insights into the factors that influence the consistency rate of genotype calls across reference genome versions, which can inform future studies on the topic.

## Results

### Web application “SoyDBean”

**Figure 6:**
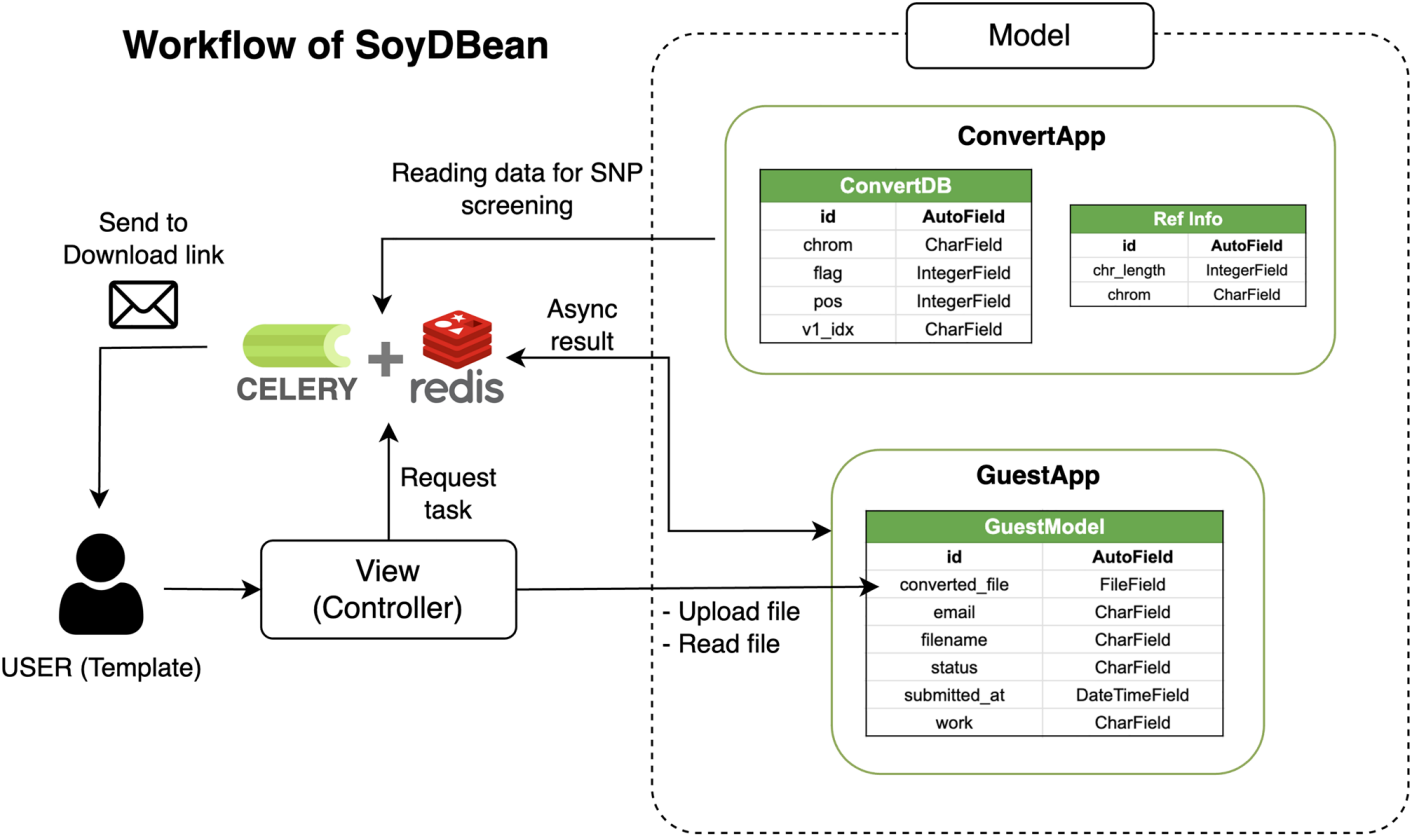
Web application "SoyDBean". Data Flow Diagram (DFD) and Entity Relationship Diagram (ERD) of the application and web framework models. The data conversion process asynchronously retrieves information from models. When the asynchronous operation is finished, the link to receive the result file is sent to the entered email. If the researcher registers on SoyDBean, Django-Models include a model for screening VCF data and a model that records the screening process.

As a method to utilize our reliable SNP database, we developed a web application, “SoyDBean” using Django (version 3.25, https://www.djangoproject.com/) and Bootstrap (version 5.2, https://getbootstrap.com/). Users can upload a VCF file of versions Glyma 1.01 and Wm82.a2.v1, and the web interface will select and update input SNPs to the reliable SNPs contained in the database (Fig. 6). All tasks are accomplished in an asynchronous manner, and the result containing a download link can be sent via email. It usually takes up to 15 min for the full conversion of SNP positions. Additionally, the web application allows users to download the SNP set confirmed by pair-wise mapping methods, which can be used for base quality score recalibration.

### Verification with a multi-sample VCF accessible to the public

**Figure 7:**
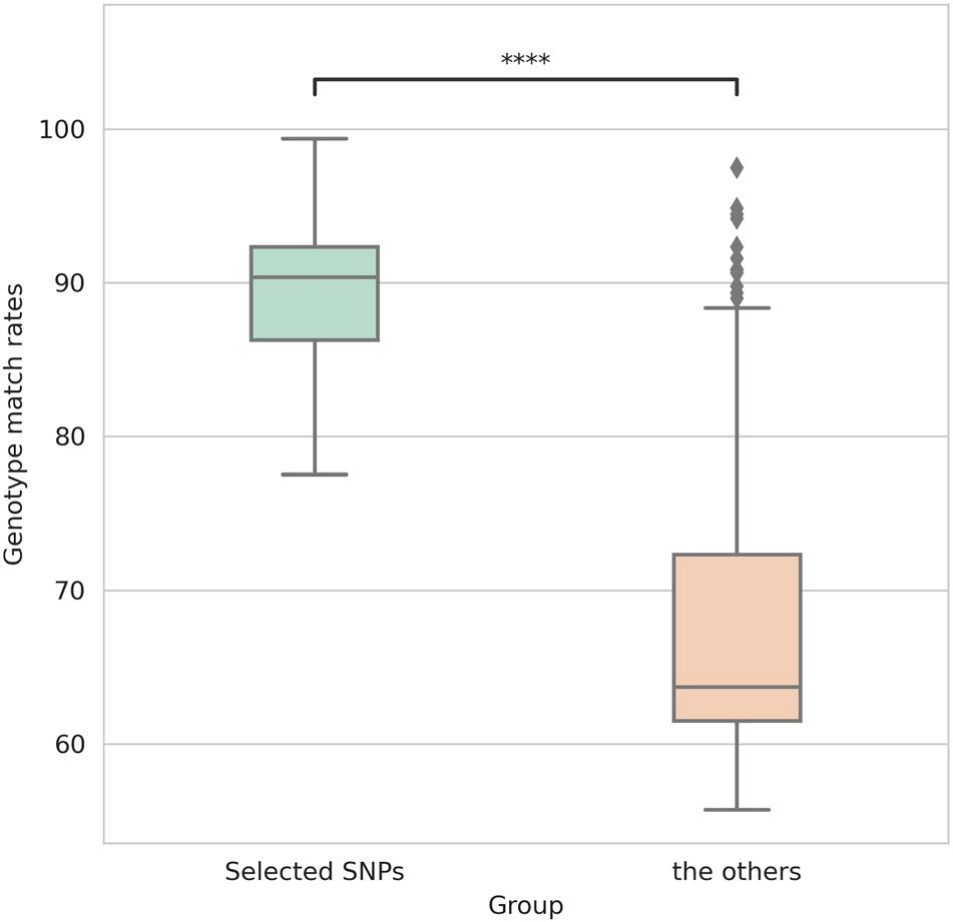
Boxplots for genotype match rates of two groups of SNPs analyzed in our study. Selected SNPs were screened from our datasets, while the others included the remaining SNPs that passed the mapping stage but were excluded during genotype match rates filtering. ****p ≤ 0.0001.

**Figure 8:**
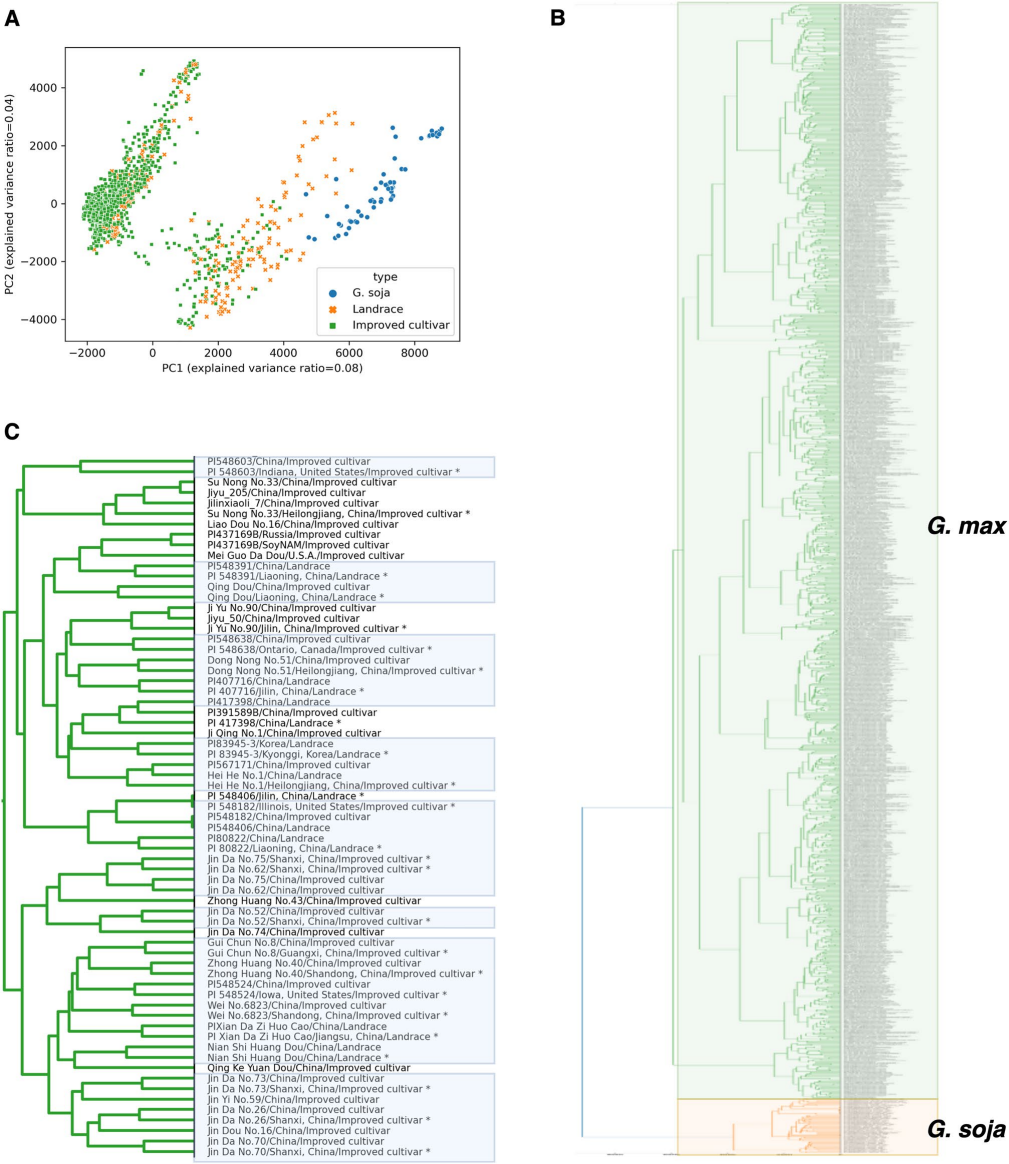
Clustering of genotype matrix using PCA. The asterisk represents a accessions that are commonly shared. (**A**) PCA plot of the first two components for 1309 accessions (1005 Improved cultivars, 242 Landrace, and 62 wild soybeans). (**B**) Phylogenetic tree of the 1309 samples. The green area indicates *G. max* and the orange area indicates *G. soja*. (**C**) Enlarged graph of a portion of the *G. max* region. The area marked in blue includes accessions classified within the same cluster.

To ensure the reliability of our SNP dataset, which was selected through pair-wise reads mapping and genotype match rates, we took the approach of consolidating publicly accessible VCFs from Soybase^27^ into a unified genotype matrix. We attempted to combine the VCFs that were based on different references, including VCF written in Glyma 1.01 (^8^, 62 wild, 130 landraces, and 110 improved cultivars; containing 9,683,313 SNPs), VCFs written in Wm82.a2.v1 (^11^, 1007 cultivated soybeans, sharing an intersection with 518 cultivated soybean accessions in above VCF^8^; containing 12,197,920 SNPs). This enabled us to generate a matrix with 4,135,736 SNPs from 1309 accessions (1005 cultivated soybean, 242 landraces, and 62 wild), excluding SNPs for which genotype information was missing more than 5% of 1309 accessions in the VCFs integration process. Firstly, we grouped the SNPs screened from our datasets, and the remaining SNPs (which passed the mapping stage but were excluded during genotype match rates filtering), and examined their genotype match rates in 1309 accessions consolidated matrix (Fig. 7). We observed that the median genotype match rate (of 518, there are common accessions in both VCFs) for 4,135,735 selected SNP positions was 90.38%, while the median genotype match rate for 10,348 remaining SNP positions was found to be 63.7%. This implies that not only the mapping process but also the comparison of genotypes is necessary to identify more reliable positions. Secondly, we utilized Principal Component Analysis (PCA) to verify that the genotype matrix was condensed in a manner that preserved the primary features (with a sum of the explained variance ratio of 0.95, n_components = 833). After plotting the first two components of the 1309 accessions, it was determined that the cultivated and wild soybeans were still able to be accurately distinguished, even after the reduction of SNPs during the SNP and merge processes (Fig. 8A). To validate our genotype matrix further, we used clustering method to classify each accession into a group and verified the results using the accession’s metadata (Fig. 8B). Our results showed that the same accessions from various datasets were grouped into the same cluster (Fig. 8C).

## Discussion

As sequencing technology continues to advance and costs decrease, there is a growing abundance of whole genome variant calls for *G. max* that is now accessible in public databases. However, due to the lack of a universally agreed-upon gold standard for generating variant data, there are still limitations in comparing and analyzing these large-scale datasets with individual data. Hence, we suggest that an effective approach for the selection of reliable SNPs would involve the comparison of genome sequences and genotypes across numerous samples, utilizing mutual reference genomes. To test this hypothesis, we employed mapping and genotype calling methods between reference genomes, which enabled us to identify comparable and dependable SNP positions, even when the reference genome changes. As a result, we utilized a reliable set of SNPs to combine Variant Call Format (VCF) from different reference genomes that had already been published and obtained a large and robust dataset that can be utilized for further molecular market-based studies^28^. In addition, we ascertained that the composition of the reference sequence is vital for the reliability of SNPs, such as the extent to which the quantity of K-mers containing "C" and "G" is high or low. It has been reported that high GC-content and repeat-rich regions are associated with assembly errors, especially in short reads^29^, which can potentially affect the reliability of genotype calls. As reference genomes have improved, they cover a greater portion of the genome, but challenges still remain in SNP validation. Therefore, we adopted a conservative approach and generated a reliable SNP dataset through mutual comparisons with each reference genome. We conducted a comparison between our reliable SNP dataset and the 170,223 SNPs that had been previously selected for the creation of the 180K AXIOM® SoyaSNP array^30^. This analysis revealed that 73% of these SNPs (132,319 SNPs) were found within our reliable SNP dataset. By refining the SoyaSNP array using our dataset, we can enhance the reliability of SNP array analyses. Furthermore, we plan to incorporate the updated soybean reference genome (Wm82.a4.v1) into our efforts to further update the set of reliable SNPs.

Currently, our database was created using 167 accessions of cultivated and wild soybeans. However, as we continue to add more datasets to our pipeline, the number of reliable SNPs is expected to increase. We are currently incorporating additional datasets and plan to update our pipeline on a quarterly basis to ensure the inclusion of the latest available data. The “SoyDBean” database has been created to enable researchers to filter and process data that has been built using different pipelines and reference genomes, into set of comparable and reliable SNPs. Furthermore, our database offers a *G. max* SNP dataset that researchers can employ in the BaseRecalibrator step of the GATK pipeline for SNP validation tools. This allows for the calibration of the sequencing data, improving the accuracy and reliability of downstream analyses. By incorporating the reliable SNPs dataset in the calibration process, researcher can reduce systematic errors in the data and improve the quality of our analysis results. This dataset can be accessed at http://soydbean.plantprofile.net/downloads/. Our approach will facilitate the analysis and integration of published variant data, thereby providing researchers with access to a wider range of variation information from various populations. This is expected to increase the likelihood of identifying genomic locations that are associated with desired characteristics, thereby aiding the genome-based breeding process.

### Supplementary Information

Supplementary Information 1.

Supplementary Information 2.

## Data Availability

The generated data and tools can be accessed through the web database “SoyDBean”, which is available at http://soydbean.plantprofile.net/.
