# Supplementary figures and images for: SoyDBean: a database for SNPs reconciliation by multiple versions of soybean reference genomes
Source: Sci Rep. 2023 Sep 21;13:15712. doi: 10.1038/s41598-023-42898-1 (PMC10514325; doi:10.1038/s41598-023-42898-1)

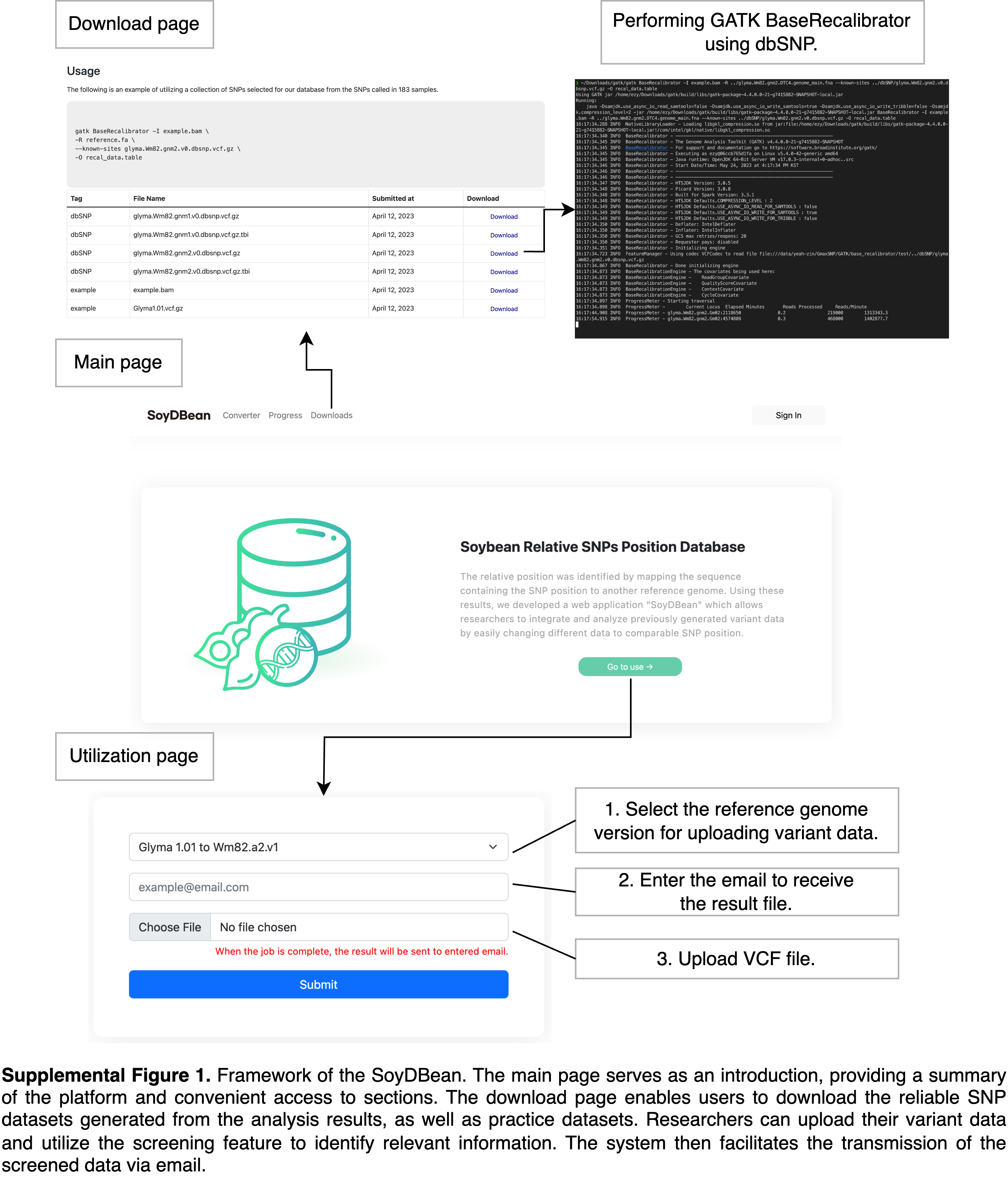

Supplement: Supplementary file 2 — Supplementary Information 2. [file 41598_2023_42898_MOESM2_ESM.png]
